# The Effects of Home Language and Bilingualism on the Realization of Lexical Stress in Welsh and Welsh English

**DOI:** 10.3389/fpsyg.2019.03038

**Published:** 2020-01-22

**Authors:** Ineke Mennen, Niamh Kelly, Robert Mayr, Jonathan Morris

**Affiliations:** ^1^Department of English, University of Graz, Graz, Austria; ^2^Department of English, American University of Beirut, Beirut, Lebanon; ^3^Centre for Speech and Language Therapy and Hearing Science, Cardiff Metropolitan University, Cardiff, United Kingdom; ^4^School of Welsh, Cardiff University, Cardiff, United Kingdom

**Keywords:** lexical stress correlates, linguistic experience, language contact, bilingualism, Welsh, Welsh English

## Abstract

This study investigates effects of long-term language contact and individual linguistic experience on the realization of lexical stress correlates in Welsh and Welsh English. To this end, a production study was carried out in which participants were asked to read out Welsh and English disyllabic words with stress on the penultimate syllable, placed within carrier phrases. Recordings were made of the productions of Welsh and English target words, by two groups of Welsh-English bilinguals differing in home language, as well as the productions of English target words by Welsh English monolinguals and speakers of Southern Standard British English (SSBE). Acoustic measures were taken of fundamental frequency (f0) and intensity ratios of stressed and unstressed vowels, duration of stressed and unstressed vowels, and duration of the post-stress consonant. The results of acoustic comparisons of Welsh English with SSBE and Welsh revealed that SSBE differs from the other groups in all measures of lexical stress. Welsh and Welsh English, however, show considerable phonetic overlap, albeit with language-specific differences in two of the five measures (unstressed vowel duration, intensity ratio). These findings suggest cross-language convergence in the realization of lexical stress in Welsh and Welsh English disyllabic words with penultimate stress. Individual linguistic experience, in turn, did not play a major role in the realization of lexical stress in these words. Bilinguals did not differ from monolinguals when speaking English, and home language also had no effect on any measure. This suggests that other factors must be responsible for the observed patterns. We discuss the possibility that the varieties of Welsh and Welsh English spoken in this community function as a sign of regional or peer group identity, rather than as markers of linguistic experience.

## Introduction

A common by-product of bilingualism is that of cross-linguistic interaction (also sometimes referred to as interference or transfer), where features from one language are transferred to or influence the other language (see Treffers-Daller and Mougeon, 2005, for a discussion). In bilingual speech production, such cross-linguistic interaction commonly results in a ‘merging’ of the phonetic properties of the first (L1) and second (L2) language, with phonetic values that are often intermediate between the monolingual values of the two languages (as evidenced by studies of segmental, e.g., Flege and Eefting, 1987; Flege, 1995; Kehoe, 2002, as well as prosodic aspects of speech production, e.g., Queen, 1996; Elordieta and Calleja, 2005; Mennen et al., 2014). This phonetic divergence from monolinguals has been attributed to a number of extra-linguistic factors. One of these is age of acquisition (e.g., Asher and Garcia, 1969; Flege et al., 1999; Piske and MacKay, 1999), either due to maturational constraints (an age-related loss of neural plasticity which causes declines in language learning ability, c.f. Long, 1990; Højen and Flege, 2006) or because it may interact with other factors such as chronological age or amount of L1 and L2 use (c.f. Flege et al., 1997; Flege, 2007).

Most research examining the influence of extra-linguistic factors has focused on those who have acquired an L2 later in life while residing in an L2-speaking environment (see Colantoni et al., 2015, and Piske et al., 2001 for reviews) or on heritage language speakers (e.g., McCarthy et al., 2013, 2014; Nagy, 2015; Lein et al., 2016; Mayr and Siddika, 2018), while the factors influencing speech production in *simultaneous* or *early bilingual speakers* residing in areas with long-standing *societal* or *regional bilingualism* have received far less research attention. The few available studies suggest that extra-linguistic factors may also influence phonetic variation among simultaneous or early bilingual speakers. In particular, the factors age of acquisition and language dominance have been shown to influence speech production in bilingual societies. Guion (2003), for instance, investigated the ability of four groups of Quichua-Spanish bilinguals to acquire Spanish vowels. All bilinguals acquired Quichua as their L1 and Spanish as their L2, but the four groups differed in the age at which they acquired the L2. The results showed a clear effect of age of acquisition: all simultaneous bilinguals (i.e., those who learned both languages from birth), most ‘early’ bilinguals (i.e., those bilinguals who began learning Spanish between the age of 5 to 7), and half of the ‘mid’ bilinguals (who began learning Spanish between the age of 9 to 13) were able to produce the cross-language vowel differences, whereas all but one of the late bilinguals (who began learning Spanish after the age of 15) were unable to do so. Furthermore, those early bilinguals who were able to produce the cross-language vowel distinctions, were found to also differ from the late acquirers in their production of L1 Quichua vowels. This suggests that a bilingual speaker’s two languages can mutually influence one another.

Simonet (2010, 2011b, 2014) carried out a number of studies investigating the role of language dominance on speech production of Catalan-Spanish early bilinguals. These studies showed that in each language the productions of /l/ (Simonet, 2010) and of the Majorcan Catalan/o/-/Ɔ/vowel contrast (Simonet, 2011b, 2014) differed according to the language dominance of the bilingual speakers. While some of the Spanish-dominant bilinguals (with home language Spanish) merged (Simonet, 2011b) or partially merged (Simonet, 2014) both vowel categories as well as /l/ (Simonet, 2010), the Catalan-dominant bilinguals (with home language Catalan) differentiated the two languages on these measures. It was suggested that “the phonetic manifestation of the contrast differed as a function of linguistic experience” (Simonet, 2014, p. 36) and that socio-indexical reasons may be at the core of the lack of these contrasts in the Spanish-dominant bilinguals (Simonet, 2010, p. 676). An influence of language dominance was also found in two studies of Galician mid vowels (Amengual and Chamorro, 2015; Tomé Lourido and Evans, 2018). Both found that only Galician-dominant bilinguals distinguished Galician-specific front and back mid vowel categories, while Spanish-dominant bilinguals, and in the case of Tomé Lourido and Evans (2018) also Galician new speakers, so-called *neofalantes*, largely merged them. In contrast, Mayr et al. (2019a) reported merged mid vowel categories even for Galician-dominant bilinguals from Galician-speaking homes with continuous high use of the language. The authors argued that in addition to cognitive factors, neutralization is likely a result of inconsistent input and socio-indexical factors.

The above studies all examined the influence of extra-linguistic factors on *segmental* aspects of bilingual speech production. While *prosodic* aspects of bilingual speech production have received some research attention (e.g., Queen, 2001; Elordieta, 2003; O’Rourke, 2003; Colantoni and Gurlekian, 2004; Lléo et al., 2004), the extra-linguistic factors influencing bilingual speakers’ prosodic productions have been examined to a much lesser extent. To our knowledge, only two papers can shed light on the role of extralinguistic factors in the production of prosody in early bilingual speakers residing in an area with long-standing societal or regional bilingualism. Simonet (2011a) investigated the intonation patterns of 10 Catalan-dominant and 10 Spanish-dominant bilinguals who were all long-term residents in Majorca. His results showed that the language dominance (determined on the basis of a language background questionnaire) and language proficiency (determined by means of a global foreign accent rating task) of the speakers affected the bilinguals’ ability to produce cross-language differences in intonation between Catalan and Spanish. In particular, his study showed that the Catalan-dominant bilinguals were more likely to differentiate the intonation patterns of the two languages than the Spanish-dominant bilinguals, who had a tendency to transfer the intonation patterns of their dominant language (i.e., Spanish) into their non-dominant language (i.e., Catalan). Ordin and Mennen (2017) examined the production of pitch range in the speech of simultaneous Welsh-English bilinguals. Although their study did not specifically set out to test the role of language dominance on the production of pitch range, a qualitative inspection of their data suggested that the English-dominant bilinguals from English-speaking homes who followed English medium education had a tendency to differentiate pitch range in their two languages, whereas the Welsh-dominant bilinguals did not. This pattern was, however, only observed in the female participants of their study, which was interpreted by the authors as resulting from the need for men “to manifest their individuality,” which they argued was less strong in women who were more likely “to conform to the society norms” in each language (Ordin and Mennen, 2017, p. 1502). These two studies suggest that prosodic variation may be influenced by similar factors as those observed in segmental aspects of bilingual speech production.

Cross-linguistic interaction is, however, not only apparent in individual bilingualism but can also occur in situations of language contact. Research has shown that such cross-linguistic interaction or transfer may over time lead to contact-induced language change (e.g., Treffers-Daller and Mougeon, 2005). A great number of factors have been shown to determine contact-induced change, amongst others the relative prestige of the languages, the relative social status of the speakers, and the degree of bilingualism across the communities in contact (Thomason and Kaufman, 1988). The long-term effect of contact-induced language change may be convergence (Backus, 2004, p. 179), which, according to Thomason (2001, p. 262), describes “a process through which two or more languages in contact become more similar to each other […] when both or all of the languages change.”

Studies of contact varieties have documented many cases where the sound systems of languages in contact have become, or are becoming, more similar to one another (Campbell and Muntzel, 1989; Heselwood and McChrystal, 1999; Bullock and Gerfen, 2004; Colantoni and Gurlekian, 2004; Louden and Page, 2005; Chang, 2009; Heeringa et al., 2015; Kirkham and Nance, 2017; Schoormann et al., 2017). Although any aspect of the sound system – including its prosody – can show convergence (Thomason and Kaufman, 1988; Winford, 2003), most studies have focused on segmental changes. Fewer studies have investigated to what extent prosody is prone to contact-induced changes, as the majority of studies have focused on contact-induced changes in intonation patterns (e.g., Queen, 2001, for Turkish and German; O’Rourke, 2005, 2012 and Muntendam and Torreira, 2016, for Quechua and Spanish intonation; Bullock, 2009, for English and French intonation; Colantoni and Gurlekian, 2004 and Colantoni, 2011a, for Italian and Spanish intonation; Elordieta, 2003; Elordieta and Calleja, 2005; Elordieta and Irurtzun, 2016, for Basque and Spanish intonation; Simonet (2011a) and Romera and Elordieta, 2013, for Catalan and Spanish intonation; Colantoni, 2011a, for Guarani and Spanish). Other prosodic aspects of speech, such as stress or rhythm, have received far less attention (Bullock, 2009; Colantoni, 2011b). Furthermore, the role of linguistic background has largely been ignored in these studies (except for Simonet, 2011a, who examined the role of home language, as described above).

This study attempts to add to the existing literature on cross-language interaction in a particularly interesting sociolinguistic context in West Wales, where Welsh-English bilingual speakers live alongside monolingual speakers of English in a language-contact situation. In particular, this study aims to investigate the role of linguistic background (often conceptualized as home language or age of acquisition) in both Welsh and Welsh English (the term used to describe varieties of English in Wales which are influenced, albeit to varying degrees, by contact with the Welsh language, see Thomas, 1994).

The work also contributes to recent work which examines variation in the speech of Welsh-English bilinguals. Morris (2013, 2017) examined variation in the production of /r/ and /l/ in the Welsh and English of school-aged bilinguals from different areas in North Wales. Having considered the influence of a number of linguistic and extra-linguistic factors on variation, he found that home language was a particularly strong influence on the pronunciation of /r/ in both languages in Caernarfon, a town where most bilinguals are from Welsh-speaking homes. He suggests that home language differences may be explained by social rather than cognitive effects in this case, as home language had social significance among the Caernarfon peer group in his study and was subject to complex interactions with speech context (sociolinguistic interview) and speaker gender (Morris, 2013, p. 261). Specifically, participants’ home language (either Welsh or English) was a defining aspect of peer-group membership and inherently linked to attitudes and use of the Welsh language in Caernarfon. In English-dominant areas, however, home language held less importance among young speakers and the language of peer-group interaction outside of the Welsh-medium classroom was English (see Morris, 2014 for an overview). Similar to Ordin and Mennen (2017), female speakers in Caernarfon tended to conform to standard pronunciation in Welsh (that is to say, the production of the alveolar trill). Such differences may be specific to individual linguistic features, however, as no home language differences were found in relation to /l/ -darkening. Instead, it was found that speaker gender influences the production of /l/ with female speakers being more likely to differentiate between the two languages (Morris, 2017, p. 199).

The assertion that social factors, such as peer group identity, may override the effects of linguistic experience on speech production is also found in Mayr et al.’s (2017) study of monophthong production based on the same speakers included in the current study. Despite comparing bilingual speakers from both Welsh and English-speaking backgrounds and English monolinguals, and controlling for phonetic context, they found no between-group differences in vowel realization within Welsh and English, although there were some differences across the languages. These results were interpreted as arising from speakers with different linguistic backgrounds being members of the same adolescent peer groups. Similar to the English-dominant area discussed by Morris (2014), participation in such a close-knit social structure where speakers’ home language had little social meaning appeared to have a homogenizing effect on vowel realizations (see also Nance, 2019 for similar results in a Gaelic-medium immersion school setting).

Finally, Mayr et al. (2019b) examined whether monolingual speakers of Welsh English and Welsh-English bilinguals from the same area in South West Wales could be distinguished solely on the basis of their English accent. In this study, the speakers were of similar ages but were sought from different areas and did not form a coherent peer group. The results of an accent perception experiment revealed that although identification accuracy was above the 50% chance level, performance was unexceptional, in particular on monolingual samples with average accuracy scores of merely 59%. The authors interpreted listeners’ difficulties as arising from the combined effects of long-term language contact effects in the speech of monolinguals and individual bilingualism effects in the speech of bilinguals, which resulted in highly subtle differences in accentual patterns. As part of the study, listeners were also asked to comment on features associated with both bilingual and monolingual Welsh English speech. The results revealed that listeners had a much clearer notion of what constitutes a bilingual’s than a monolingual’s accent, with the former being mostly perceived as containing features derived from Welsh, such as trilled realizations of /r/. The monolinguals’ accents, in turn, were associated with the absence of these features as well as with greater use of vernacular forms that are not limited to Welsh English, such as /t/ -glottalling, /h/ -dropping or the use of alveolar nasals instead of velar nasals for (ing).

Despite this previous work, little is known about the extent to which the speech production of bilingual speakers residing in areas with long-standing societal or regional bilingualism is influenced by linguistic background, and even less is known about prosodic features of speech production. Therefore, this study investigates a prosodic feature that has received very little research attention, namely the production of lexical stress. The inclusion of bilingual and monolingual speakers residing in the same area made it possible to investigate the effects of both individual linguistic experience (conceptualized here as home language and bilingualism) and long-term language contact on the production of lexical stress in this community. In order to achieve these aims, we sought answers to the following questions:

(1) How do monolingual speakers of Welsh English realize lexical stress in comparison to speakers of Welsh and of Southern Standard British English (SSBE)?
(2) Do monolinguals and bilinguals from the same community differ in their production of the lexical stress correlates when speaking English?
(3) To what extent does home language influence variation in lexical stress productions?

### Lexical Stress in Welsh and English

Welsh and English can both be categorized as lexical-stress languages (e.g., Cutler, 2005). That is, in words with more than one syllable, there is usually one syllable that stands out and is perceived as more prominent than others. English is a free-stress language in which the location of stress is unpredictable and varies across words. In free-stress languages, stress can be used to distinguish between otherwise identical words, as in the noun SUBject and the verb subJECT (upper case is used to refer to the stressed syllable) (Fry, 1955). Welsh, on the other hand, is a fixed-stress language, where the location of stress in a word is largely predictable, and usually falls on the penultimate syllable regardless of the number of syllables in the word (e.g., Williams, 1999; Ball and Williams, 2001; Hannahs, 2013), as in words like ‘blynyddoedd’ [blә^|^nәðƆIð] *years*, and ‘cwmwl’ [^|^kʊmʊl] *cloud* (examples taken from Czerniak, 2015). Irregular stress patterns in Welsh mostly have stress on the final syllable, as in ‘canŵ’ [ka^|^nuː] *canoe*, or less commonly on the antepenultimate syllable, as in ‘paragraff’ [^|^paragraf] *paragraph* (examples from Czerniak, 2015). These irregular stress patterns are rare and occur predominantly in loanwords from English (Williams, 1983; Ball and Williams, 2001).

Lexical stress can be expressed by a combination of acoustic correlates, such as fundamental frequency (f0), intensity, duration and vowel quality. A number of studies have shown that these correlates and their relative importance in both production and perception may differ across languages (Beckman, 1986; Beckman and Edwards, 1994; Sluijter and van Heuven, 1996; Cutler, 2005). Most studies on the relative importance of acoustic correlates of English lexical stress have compared primary stressed syllables with unstressed syllables in words produced in isolation or in focus. In such circumstances, the primary stressed syllable is also accented and carries a pitch movement, whereas the unstressed syllable is unaccented (Sluijter and van Heuven, 1996). Under such conditions (and the conditions adopted in our study), the most important and reliable acoustic correlates of English lexical stress are vowel duration, f0, and intensity. Thus, primary stressed syllables in English are longer, louder, and have higher pitch than unstressed syllables (Fry, 1955; Lieberman, 1960; Beckman, 1986; Beckman and Edwards, 1994; Campbell and Beckman, 1997). Under conditions where stressed syllables are not also accented, the most reliable acoustic correlate of stress is duration and spectral balance, whereas f0, intensity and vowel quality play a less reliable role. The acoustic correlates of lexical stress in Welsh are, on the other hand, conspicuously different from those in SSBE. It has long been noted that stress in Welsh is unusual in that it “is not directly related to the usual acoustic cues of f0, intensity and duration of the stressed vowel” (Williams, 1985, p. 381). For instance, early auditory observations of Welsh (e.g., Jones, 1949; Watkins, 1953; Oftedal, 1969) note that stressed syllables do not necessarily feature greater amplitude, longer duration or higher pitch than the following unstressed syllable, that the difference between the stressed penult and unstressed ultima is rather small, and that post-stress consonants (PSCs) often have extra duration. Instrumental confirmation of these auditory observations comes from a series of studies by Williams (1983, 1985, 1999) and Ball and Williams (2001) which systematically investigate the acoustic correlates of lexical stress production and perception in Welsh. These studies show that Welsh stressed syllables do *not* have the typical SSBE stress cues of longer vowels, a higher f0 or higher intensity *when compared to unstressed syllables*. Furthermore, unlike English, vowel reduction is not a main stress cue in Welsh^1^. When compared to unstressed vowels, Welsh stressed vowels may have a higher or lower pitch, greater or smaller amplitude, and may be equally long (Williams, 1985; Ball and Williams, 2001). Instead, the PSC (the consonant immediately following the stressed vowel) is lengthened. These differences between Welsh and English are supported by the fact that listeners appear to use their native language acoustic cues to judge the position of stress (Williams, 1983, 1985). When listening to Welsh words and sentences read aloud, English speakers judge vowels as stressed when they feature a greater f0 change, longer duration, and higher intensity. Welsh listeners, on the other hand, use the duration of the PSC as the primary cue to judge vowels as stressed.

Very little is known about the realization of lexical stress in Welsh English. Some descriptive accounts report that Welsh English shares some of the acoustic correlates of stress with Welsh. For instance, Walters (2003) observes that one device used by speakers of Rhonda Valley English (South Wales) “to help impart a strong accent is, instead of lengthening the stressed vowel, to markedly shorten it and lengthen the succeeding consonant” (p. 218). He goes on to say that “[a]n even more striking feature is that the post-stress syllable may be as strong phonetically as the stressed one, with as great (or greater) intensity and duration, and higher pitch that carries much of the pitch movement associated with the accent” (p. 220). Connolly (1981) reports that Welsh English spoken in Port Talbot (South Wales) also features a lengthening of the PSC, and attributes this to an influence from Welsh. To our knowledge, only one instrumental study has investigated the realization of lexical stress in Welsh English (Webb, 2011). However, the study only focused on durational aspects of lexical stress, and ignored other acoustic correlates. Specifically, Webb (2011) compared segment durations of six Welsh-English bilingual females from North Wales to five monolingual female SSBE speakers. The results showed that although the realization of stress in the English spoken by the Welsh-English bilinguals showed some similarities to Welsh, it is not the same and “seems to lie somewhere between SSBE and Welsh” (p. 2109). In terms of the duration of the PSC, Welsh English was found to be more similar to Welsh. The duration of the unstressed vowel, however, was found to be closer to SSBE. It remains unclear whether the reported differences are a result of a historical influence of Welsh or whether they are due to the interaction of the two languages of bilingual speakers, given that no comparisons were made with monolingual English speakers who live in the same community.

To summarize, previous studies have shown that Welsh and SSBE differ greatly in their acoustic realization of lexical stress. However, surprisingly little is known about the realization of lexical stress by monolingual speakers of the contact variety Welsh English, and there are no studies on the role of home language in the production of lexical stress cues by Welsh-English bilingual speakers. While it is often reported that Welsh English shares some of the acoustic correlates of stress with Welsh, it is also suggested that some acoustic correlates may be realized at some intermediate point between Welsh and SSBE. In order to shed light on this issue, our study also included a group of monolingual speakers of SSBE. This study then aims investigate lexical stress produced by monolingual and bilingual speakers from the same community and attending the same bilingual school in Carmarthenshire, South West Wales. Two groups of bilinguals are investigated: one with Welsh as their home language, the other with home language English (see further in the next section). The inclusion of two groups of bilinguals makes it possible to investigate the role of linguistic background in the production of lexical stress.

## Materials and Methods

### Participants

There were 40 male participants in this study (*n* = 10 per group)^2^ ^,3^. Thirty males were from the town of Ammanford in Carmarthenshire (South West Wales), where 32.9% of the population is able to speak, read and write Welsh (Welsh Government, 2013). They were all between the ages of 16 and 18 at the time of recording, and attended the same local bilingual secondary school. In this school, pupils can choose to either follow the curriculum wholly in English (with the exception of Welsh as a Second Language), or they can follow the majority (up to 80%) of their teaching through the medium of Welsh. Although pupils who follow the Welsh-medium pathway typically study for statutory examinations in Welsh as a First Language, they may differ in their experience with the Welsh language. Some will have acquired it from birth and are from Welsh-speaking homes; others will have acquired it through the medium of education and are from English-speaking homes. Those pupils following the English-medium pathway typically have little functional knowledge of Welsh, although they have compulsory Welsh as a Second Language classes until the age of 16.

The 30 male adolescents from Ammanford were assigned to one of three groups based on their language experience. The participants in the first group, i.e., L1WEL, were Welsh-English bilinguals who came from Welsh-speaking homes (where both parents spoke Welsh; *n* = 10) and followed the Welsh-medium pathway. The participants in the second group, i.e., L1ENG, were also Welsh-English bilinguals, but came from English-speaking homes (where both parents spoke English; *n* = 10) and had acquired Welsh solely via immersion education (i.e., they also followed the Welsh-medium pathway). Participants of the third group, i.e., MONOENG, were English monolinguals from English-speaking homes (*n* = 10) and followed the English-medium pathway. None of the participants in this group reported more than a very rudimentary knowledge of Welsh, and they typically reported being unable to hold a conversation in Welsh. Despite having been taught in different classes for much of their education, the participants shared social areas and reported that the main language of interaction was English.

Further information on bilingual participants’ language use was collected via questionnaire during the data collection. Participants were asked to specify the amount of time they used Welsh in specific contexts expressed as a percentage (see also Mayr et al., 2017). As shown in Table 1, there are clear differences in the language use patterns with parents of siblings of the L1WEL and L1ENG groups. Despite some individual variation among participants from the L1WEL group, the main language used in contexts outside of the family tended to be English.

**TABLE 1:** Percentages of self-reported weekly use of Welsh amongst bilingual participants from Welsh-speaking (L1WEL) and English-speaking (L1ENG) homes.

Speaker	% Parents	% Siblings	% School friends	% Friends from outside school	% In the community	% Media
W1	80	100	5	0	10	25
W2	80	70	20	0	0	5
W3	100	50	0	0	0	0
W4	100	100	0	0	0	25
W5	80	50	0	0	0	0
W6	95	90	0	0	50	20
W7	100	N/A	10	20	10	10
W8	90	N/A	0	0	50	0
W9	100	100	20	25	5	5
W10	80	50	30	55	0	40
E1	0	10	0	20	0	20
E2	0	5	0	0	0	0
E3	0	N/A	5	0	0	0
E4	0	N/A	0	0	0	10
E5	0	N/A	0	5	5	10
E6	0	N/A	2	0	0	20
E7	0	50	0	0	10	10
E8	0	0	0	0	25	0
E9	0	N/A	0	0	0	20
E10	0	0	0	0	0	0

The final 10 male speakers were monolingual speakers of SSBE. The participants in this fourth group were monolingual speakers of SSBE, who were living in Southeast England and spent their formative years there. As we were unable to gain permission from secondary schools in the South of England in time for our study, the speakers of SSBE were slightly older (19–21 years) than the speakers from Ammanford (16–18 years), and had all just started university degrees. As clearly established in the literature (Williams, 1983, 1985, 1999), we would expect to find considerable differences in the realization of lexical stress correlates between Welsh and SSBE. For Welsh English speakers we would expect to find that they may share some accentual features with Welsh due to the contact situation in Wales having resulted in contact-induced change, or they may realize lexical stress at some intermediate point between Welsh and SSBE.

### Materials and Procedure

Given that Welsh has more or less fixed stress on the penultimate syllable, it is not possible to investigate lexical stress in the way we are accustomed to, i.e., by creating minimal stress pairs of the type IMport (noun) versus imPORT (verb). This is further complicated by our aim to compare stress correlates across two languages. We therefore opted for comparing pairs of disyllabic target words with stress on the penultimate syllable in which the initial syllable was the same or as similar as possible in the two languages, such as the English target word ‘melon’ /^|^mɛlәn/ and the Welsh target word ‘melyn’ /^|^mɛlIn/ *yellow*. While it is known that segments tend to become shorter as a word increases in length (Lehiste, 1972; Lindblom and Rapp, 1973), there appears to be no work specifically on whether word length interacts with the acoustic cues involved in lexical stress, likely due to the confounding effect of secondary stress. In a cross-linguistic survey of literature on the acoustic correlates of stress, Gordon and Roettger (2017) note that in experiments on secondary stress, it tends to be distinguished from primary stress by a subset of the correlates that are used to distinguish primary stress from unstressed syllables. In the current study, our goal was to focus on the difference between stressed and unstressed vowels, without the confound of secondary stress. Furthermore, we wanted to use words where the segments of the stressed syllable were matched across the two languages. In order to make this feasible and to allow us to use a high number of word pairs, we limited our study to disyllabic words.

Table 2 lists the sets of 18 target words used for English and Welsh. The majority of the 36 target words had a ‘CVCVC pattern, but some target words had a ‘CVCV pattern or in one instance a ‘CVCC pattern to allow cross-language matching of the initial syllable. The target words were embedded in a carrier phrase “Say [*target word*] again” (English); or the Welsh equivalent “Dyweda [*target word*] eto” (Welsh). As such, the target words were always in focus, as was also the case in most target words in Williams’ studies on Welsh lexical stress (1983, 1985, 1999). However, unlike in Williams’ studies, we chose to place our target words in non-final position to avoid effects of sentence/phrase final lengthening (e.g., Lehiste, 1972) and boundary phenomena (e.g., Pierrehumbert, 1980). Target words were presented twice in random order and were interspersed with materials for other experiments not reported here.

**TABLE 2:** Target words in Welsh and English.

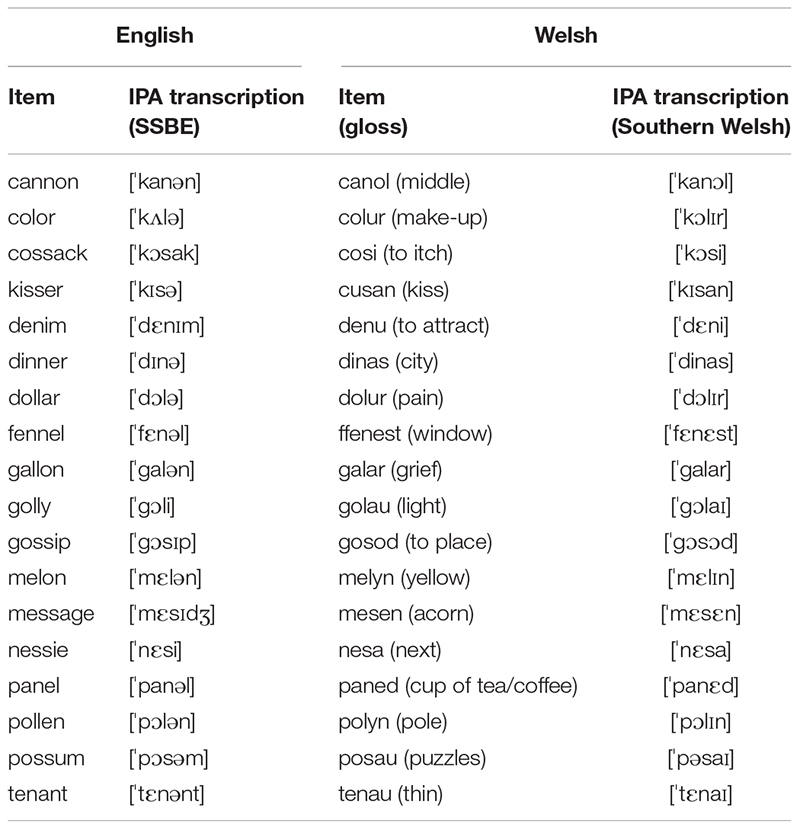

The participants were recorded individually in a quiet location at school (or at university in case of the SSBE participants) using a Zoom H2 Handy Recorder with integrated microphone at a sampling frequency of 96 kHz with 16-bit quantization. The bilingual speakers were recorded in both languages, but care was taken to control for language mode (Grosjean, 1989) by recording each language on separate days. Each individual recording took approximately 30 min per language. Participants were instructed to take as much time as needed to produce a natural reading of the carrier phrase, and to repeat any misread phrase.

### Acoustic Measurements

The first acceptable repetition was selected for further measurement, discarding those tokens that were affected by disfluencies, hesitation or noise. In those cases, the second repetition was used. This yielded a total of 353 tokens for Welsh (18 × 20 participants’ readings of Welsh tokens, with 7 tokens excluded due to poor quality) and 710 for English (18 × 40 participants’ readings of English tokens, with 10 tokens excluded due to poor quality), 1063 in total. All useable materials were subsequently annotated and analyzed using PRAAT software (Boersma and Weenink, 2016).

**FIGURE 1:**
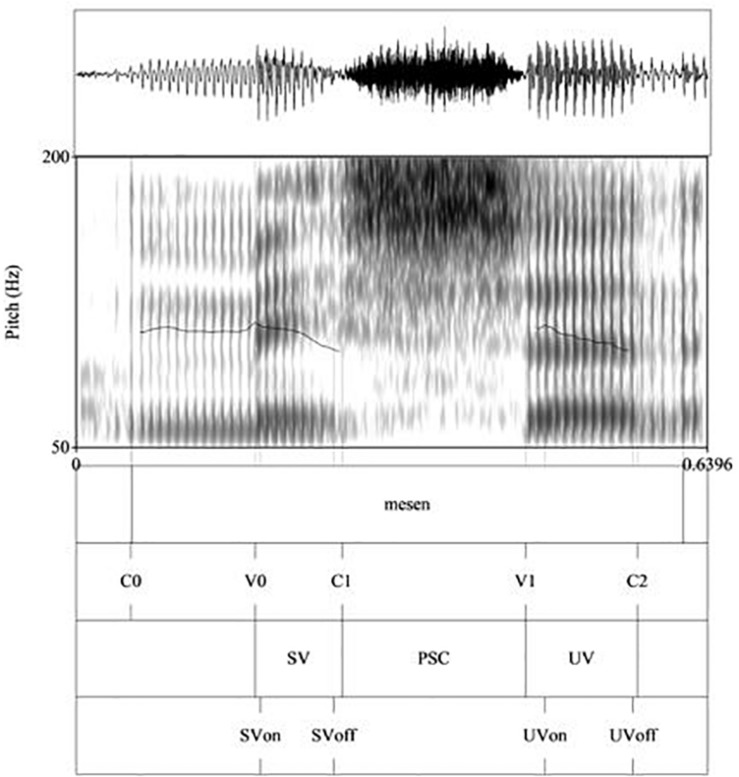
Labels and measurements points in Praat for one Welsh target word ‘mesen’ [‘ɛsɛn] *acorn*. The following labels were inserted: the onset of the word (and stressed syllable) (C0); the onset of the stressed vowel (V0); the end of the stressed vowel (C1); the onset of the following vowel (V1); and the end of the unstressed vowel (C2). From these, the durations of the stressed vowel (SV), post-stress consonant (PSC) and unstressed vowel (UV) were computed. SVon and SVoff refer to f0 at the stressed vowel onset and offset respectively. UVon and UVoff refer to f0 at unstressed vowel onset and offset.

**FIGURE 2:**
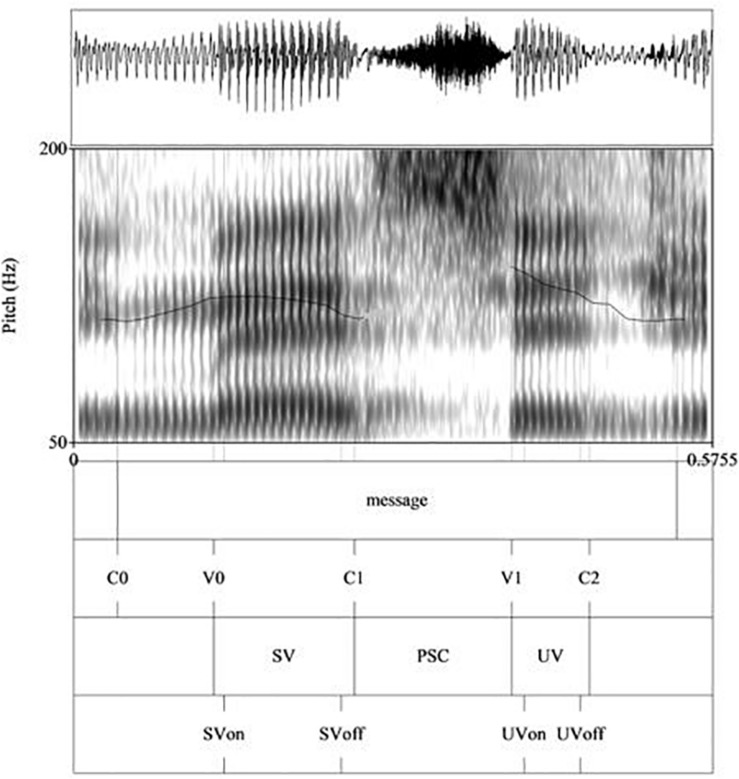
Labels and measurements points in Praat for one English target word ‘message’ [‘mɛsIdʒ]. Labels as explained in Figure 1.

Each token was hand-labeled in Praat textgrids, as in Figure 1 (showing annotations of one of the Welsh target words, ‘mesen’ [^|^mɛsɛn] *acorn*) and Figure 2 (showing annotations of one of the English target words, ‘message’ [^|^mɛsIdʒ]). The top tier shows the start and end of the target word, indicated by boundaries demarcating the start and end of the target word. The second tier shows the segmental points that were labeled following segmentation criteria described in Turk et al. (2006). These were the onset of the stressed syllable (C0); the onset of the stressed vowel (V0); the end of the stressed vowel (C1); the onset of the following vowel (V1); and the end of the unstressed vowel (C2). From these labels, measurements for the acoustic parameters of duration, intensity and f0 were taken by means of a Praat script. For duration, the following measures were computed: duration of the whole target word, the initial stressed vowel (SV), the post-stress consonant (PSC), and the final unstressed vowel (UV) (in ms), as shown in the third tier of Figures 1, 2. The latter three measures were then expressed as a percentage of the duration of the respective target word, in order to normalize for individual speaker differences. In addition, we manually marked f0 at the onset and offset of the stressed and unstressed vowels (thereby avoiding spurious f0 measures such as clear microprosodic effects or octave errors) as SVon, SVoff and UVon, UVoff respectively (as shown in the fourth tier of Figures 1, 2). In the few cases where there was a clear rise to a peak within the stressed or unstressed vowel, we instead took the peak as our SVon measure. This only applied to 28 tokens in total (19 tokens for H1, 9 tokens for H2), randomly distributed across the various groups.

We used the PRAAT standard algorithm for f0 tracking with its recommended settings for male speakers (i.e., a pitch floor of 75 Hz and a pitch ceiling of 300 Hz). Octave errors were manually corrected, whereas instances of creaky voice were excluded. For f0, we computed the average f0 across the stressed vowel and the average f0 across the unstressed vowel. We then calculated the ratio of these two (so, average of the stressed vowel divided by average of the unstressed vowel). In addition, we calculated the average f0 at the beginning and end of the stressed and unstressed vowels, in order to capture possible differences in intonation contours between the various groups of speakers. For intensity, measures of average intensity (in dB) of each stressed and unstressed vowel were taken, and the ratio of the stressed vowel intensity divided by unstressed vowel intensity was computed.

Based on previous research on Welsh and SSBE, it was hypothesized that the acoustic correlates of lexical stress in Welsh would be different from those in SSBE. Moreover, previous research on long-term contact would suggest that the acoustic correlates of lexical stress in Welsh English might be similar to those of Welsh, or intermediate between Welsh and SSBE, both for bilingual and monolingual speakers of Welsh English. Furthermore, it was expected that home language might have an effect on lexical stress, whereby the Welsh English of those from Welsh-speaking homes may have more in common with Welsh than that of those from English-speaking homes.

## Results

**FIGURE 3:**
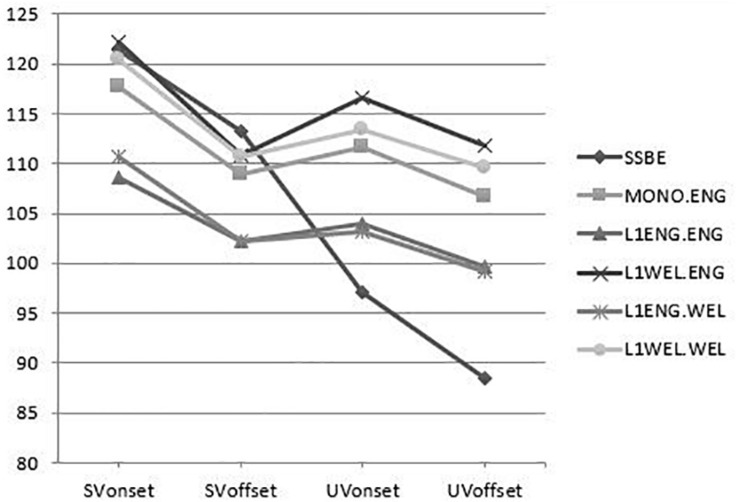
F0 values at the onset and offset of the stressed and unstressed vowels (labeled as SVon, SVoff and UVon, UVoff respectively), visualizing the intonation patterns of SSBE (the monolingual speakers of Southern Standard British English), MONO.ENG (the monolingual speakers of Welsh English), L1ENG.ENG (the bilinguals from English-speaking homes speaking English), L1WEL.ENG (the bilinguals from a Welsh-speaking home speaking English), L1ENG.WEL (the bilinguals from English-speaking homes speaking Welsh), and L1WEL.WEL (the bilinguals from a Welsh-speaking home speaking English).

In order to ensure that the participants in the various groups were not using different intonation contours, with some consistently using rising and others falling intonation, we compared the average values for f0 at the beginning and end of stressed and unstressed syllables. Figure 3 shows that while the groups differ to some extent in the relative height of the various f0 values (reflecting differences in pitch register and range), all groups produce a falling contour. Nevertheless, some differences can be observed between the SSBE speakers and the speakers in Wales in how they realized the contour. In SSBE, the stressed vowel is marked by a falling pitch accent after which f0 falls gradually from the onset of the stressed penultimate vowel to the offset of the ultima vowel (reaching an average of 89 Hz at the end of the unstressed vowel). Speakers in Wales also realize a falling pitch accent on the stressed vowel. However, unlike the SSBE speakers, this generally falling f0 pattern is interrupted by an f0 reset to a higher level at the start of the following unstressed vowel after which the contour continues to fall, but to a less low level than SSBE speakers (reaching an average of 105 Hz).

In summary, while there are some realizational differences between SSBE and the other groups, the intonation pattern is falling in all groups. Thus, our data appropriately control for any potential effect the choice of intonation contour may have on the acoustic correlates of stress (e.g., Hermes and Rump, 1994; Terken and Hermes, 2000).

Subsequently, three sets of analyses were conducted. First, we compared Welsh, Welsh English and SSBE on all measures, to examine cross-linguistic and cross-varietal differences. Next, we examined the English produced by the speakers in Wales, that is, the two bilingual groups and the monolingual English group. Finally, we directly compared the Welsh and English of the two bilingual groups, to examine the effect of home language. Together, these comparisons allow us to disentangle the roles of individual bilingualism and long-term contact on lexical stress patterns.

The statistical test used for each analysis was a multivariate linear regression, using the *lm* function in R Core Team (2016). This test shows the effect of the independent variable, *language group*, on each of the acoustic correlates of lexical stress (the measures described above). When a main effect of language group was found, and more than two groups were being compared in the model, a *post hoc* pairwise test was run using the *lsmeans* package to examine the differences between the language groups. The advantage of running a multivariate analysis is that all dependent variables can be included in one test, rather than running separate analyses for each acoustic measure. An alpha level of 0.001 was chosen, as opposed to 0.05 or 0.01, in order to reduce the chances of false positive results. The groups were coded as follows: L1WEL for speakers from Wales whose home language is Welsh, L1ENG for speakers from Wales whose home language is English, MONOENG for monolingual speakers of Welsh English, and SSBE for native speakers of SSBE. For the bilinguals, the language of the token words was encoded as ENG or WEL, such that L1ENG.WEL is a person from an English-speaking home speaking Welsh, while L1WEL.ENG is a person from a Welsh-speaking home speaking English. The reference level for the regression analysis is always the alphabetically-first independent variable, so the results in the tables are shown in comparison to that. For example, if L1ENG.ENG, MONOENG, and SSBE are compared in one analysis, the reference level will be L1ENG.ENG, and the other two variables are shown in comparison to this. The polarity of the Coefficient (β) shows the direction of the difference (positive means higher, negative means lower) and the number of the Coefficient shows the magnitude of the difference between the language groups. SE is standard error.

### Comparison 1: How Do Monolingual Speakers of Welsh English Realize Lexical Stress in Comparison to Speakers of Welsh, and Monolingual Speakers of SSBE?

In this comparison, we wanted to establish to what extent monolingual speakers of Welsh English resemble Welsh or SSBE in their realization of lexical stress. In order to establish a cross-language influence, typically comparisons are carried out with monolingual speakers of the two languages that could potentially have had an influence (cf. Meyerhoff, 2009). In our case, this was not possible (at least not for Welsh), since all adult Welsh speakers are bilingual. Therefore, in line with Mayr et al. (2017), we decided that our group of Welsh-English bilinguals with Welsh as their home language would be the closest to monolingual Welsh, as they would have had the most exposure to Welsh. We therefore compared the groups L1WEL.WEL (the bilinguals from a Welsh-speaking home speaking Welsh), MONOENG (the monolingual speakers of Welsh English) and SSBE (the monolingual speakers of SSBE).

**FIGURE 4:**
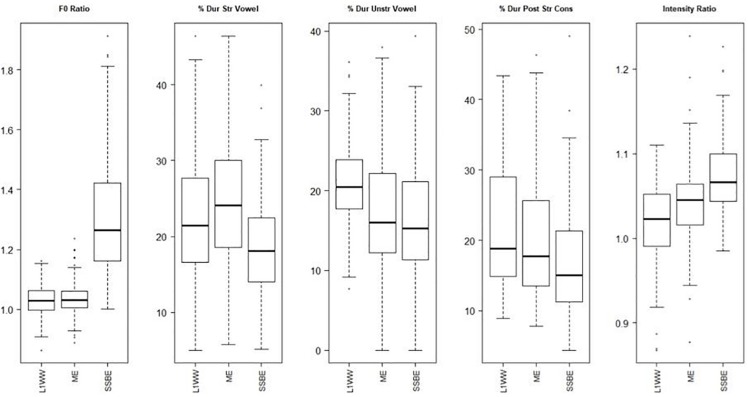
Acoustic correlates of Welsh (L1WW), Welsh English (ME), and SSBE. (% Dur Str Vowel and % Dur Unstr Vowel are the durations of the stressed and unstressed vowels, respectively. % Dur Post Str Cons is the duration of the post-stress consonant).

As shown in the boxplots (Figure 4), for the measure of f0, SSBE has a higher average f0 ratio in stressed than unstressed syllables than both Welsh and monolingual Welsh English, which appear similar to one another. These latter two show an average ratio of around 1, meaning that there is little to no difference in average f0 between stressed and unstressed syllables. In Welsh and monolingual Welsh English, the stressed vowel appears to comprise a larger percentage of the target word than in SSBE. For the duration of the unstressed vowel, Welsh English and SSBE pattern together, with the percentage of the target word being less than in Welsh. In Welsh stressed and unstressed vowels appear to comprise similar percentages of the target word, whereas in SSBE and Welsh English the stressed vowel has a higher percentage than the unstressed vowel. For the duration of the PSC, again Welsh and Welsh English pattern together, with the percentage being higher than in SSBE. Finally, the intensity difference between the stressed and unstressed vowels is highest in SSBE and lowest in Welsh. Table 3 presents the results of the statistical analysis and Table 4 shows the means for each measure by group.

**TABLE 3:** Results of the multivariate regression analysis comparing L1WEL.WEL, MONOENG, and SSBE (Dur_SV is duration of the initial stressed vowel, Dur_USV is duration of the final unstressed vowel and Dur_PSC is duration of the post-stress consonant. Each of these was measured as a percentage of the duration of the entire word, as described above).

Measure		β	*SE*	*t*	*p*
F0	Intercept MONOENG SSBE	1.030	0.009	109.952	<0.001
		0.006	0.014	0.421	0.674
		0.271	0.014	20.039	<0.001
Dur_SV	Intercept MONOENG SSBE	22.299	0.588	37.949	<0.001
		1.834	0.862	2.128	0.034
		–3.293	0.848	–3.882	<0.001
Dur_USV	Intercept MONOENG SSBE	21.034	0.512	41.047	<0.001
		–3.573	0.752	–4.755	<0.001
		–4.299	0.739	5.812	<0.001
Dur_PSC	Intercept MONOENG SSBE	21.524	0.612	35.176	<0.001
		–1.861	0.897	–2.074	0.039
		–4.95	0.883	–5.604	<0.001
Intensity	Intercept MONOENG SSBE	1.019	0.003	299.931	<0.001
		0.019	0.005	3.904	<0.001
		0.053	0.005	10.730	<0.001

*Alpha level is *p* < 0.001.*

**TABLE 4:** Mean values for each measure for Comparison 1 by language group.

Measure	L1.WEL.WEL	MONOENG	SSBE
F0 Ratio	1.030	1.036	1.307
Dur_SV	22.324	24.402	18.587
Dur_USV	21.012	17.306	16.800
Dur_PSC	21.523	20.029	16.829
Intensity	1.019	1.042	1.072

The statistical results show a significant main effect for all measures (SSBE is significantly different from the reference group (L1WEL.WEL) on all measures, while Welsh English is significantly different on two of the five measures). A pairwise analysis was conducted to determine which language groups were different from which others. These results are shown in Table 5.

**TABLE 5:** Results of the pairwise *lsmeans* tests comparing L1WEL.WEL, MONOENG, and SSBE.

Measure		β	*SE*	*t*	*p*
F0	L1WEL.WEL - MONOENG	–0.006	0.014	–0.421	0.907
	L1WEL.WEL - SSBE	–0.271	0.014	–20.039	< 0.001
	MONOENG - SSBE	–0.265	0.014	–18.938	<0.001
Dur_SV	L1WEL.WEL - MONOENG	–1.834	0.862	–2.128	0.09
	L1WEL.WEL - SSBE	3.293	0.848	3.882	<0.001
	MONOENG - SSBE	5.127	0.878	5.837	<0.001
Dur_USV	L1WEL.WEL - MONOENG	3.573	0.751	4.755	<0.001
	L1WEL.WEL - SSBE	4.299	0.739	5.812	<0.001
	MONOENG - SSBE	0.726	0.766	0.948	0.610
Dur_PSC	L1WEL.WEL - MONOENG	1.861	0.897	2.074	0.096
	L1WEL.WEL - SSBE	4.950	0.883	5.604	<0.001
	MONOENG - SSBE	3.089	0.914	3.377	0.002
Intensity	L1WEL.WEL - MONOENG	–0.019	0.005	–3.904	<0.001
	L1WEL.WEL - SSBE	–0.053	0.005	–10.730	<0.001
	MONOENG - SSBE	–0.033	0.005	–6.532	<0.001

In line with the boxplot (Figure 4, leftmost boxplot) showing the f0 difference, Welsh and Welsh English (MONOENG) show very similar patterns, while SSBE is significantly different from each of these, having a greater average f0 in stressed than unstressed syllables. In Welsh and Welsh English, the average f0 is the same across both syllables, indicating that f0 is not a cue for stress. For the duration of the stressed vowel, again SSBE is significantly different from Welsh and Welsh English. In SSBE, the stressed vowel makes up a smaller percentage of the word duration than it does in either Welsh or Welsh English, which do not differ significantly from one another. Also, as seen in the boxplots, in Welsh the unstressed vowel comprises a significantly greater percentage of the word duration than in Welsh English or SSBE, which do not differ from one another on this measure. This also shows, as already established in previous research (Williams, 1983, 1985; Ball and Williams, 2001), that in Welsh the unstressed vowel does not get shortened in comparison to the stressed vowel. Welsh and Welsh English pattern together again with their duration of the PSC, which takes up significantly more of the word duration for these two groups than it does in SSBE. Finally, the three groups are significantly different from one another in the intensity ratio. SSBE has the highest ratio (indicating the largest difference in intensity), followed by Welsh English, followed by Welsh, which differs significantly from Welsh English. Thus, for most of these measures, the results indicate that lexical stress in Welsh English has more in common with Welsh than with SSBE. For the final measure, intensity, Welsh English is intermediate between Welsh and SSBE.

### Comparison 2: Do Monolinguals and Bilinguals From the Same Community Differ in Their Production of the Lexical Stress Correlates When Speaking English?

**FIGURE 5:**
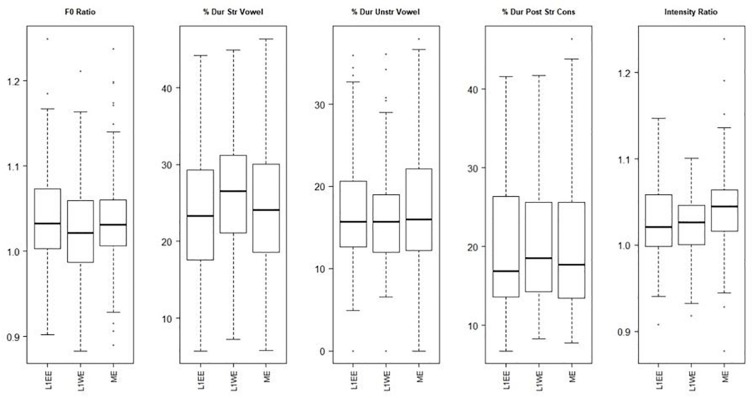
Acoustic correlates of English in Wales (L1ENG.ENG, L1WEL.ENG, MONOENG).

This analysis compares L1ENG.ENG (the group of bilinguals from English-speaking homes speaking English), L1WEL.ENG (the group of bilinguals from Welsh-speaking homes speaking Welsh) and MONOENG (the group of monolingual speakers of Welsh English), to compare the English of the bilinguals (divided by home language) and monolinguals in Wales. The boxplots (Figure 5) show that for f0 and for the durations of the unstressed vowel and PSC, there is very little difference between the three groups. The monolingual English speakers have a slightly higher intensity difference between the syllables than the two bilingual groups. Table 6 shows the statistical results and Table 7 shows the means.

**TABLE 6:** Results of the multivariate regression analysis comparing L1ENG.ENG, L1WEL.ENG, and MONOENG.

Measure		β	*SE*	*t*	*p*
F0	Intercept	1.041	0.005	224.272	<0.001
	L1WEL.ENG	–0.018	0.007	–2.606	0.009
	MONOENG	–0.005	0.007	–0.687	0.493
Dur_SV	Intercept	24.389	0.656	37.201	<0.001
	L1WEL.ENG	1.498	0.958	1.565	0.118
	MONOENG	–0.256	0.937	–0.273	0.785
Dur_USV	Intercept	16.814	0.529	31.854	<0.001
	L1WEL.ENG	–0.715	0.771	–0.928	0.354
	MONOENG	0.647	0.754	0.858	0.391
Dur_PSC	Intercept	19.870	0.645	30.812	<0.001
	L1WEL.ENG	0.732	0.942	0.776	0.438
	MONOENG	–0.208	0.921	–0.225	0.822
Intensity	Intercept	1.026	0.003	315.500	<0.001
	L1WEL.ENG	–0.003	0.005	–0.674	0.501
	MONOENG	0.012	0.005	2.499	0.013

**TABLE 7:** Mean values for each measure for Comparison 2 by language group.

Measure	L1.ENG.ENG	L1.WEL.ENG	MONOENG
F0 Ratio	1.038	1.020	1.036
Dur_SV	23.656	25.779	24.402
Dur_USV	17.061	15.932	17.306
Dur_PSC	19.693	20.719	20.029
Intensity	1.028	1.023	1.042

The regression results show that there were no significant differences for any measure. Thus, there were no significant differences on any of the measures of lexical stress among the three groups from Wales when speaking English.

### Comparison 3: To What Extent Does Home Language Influence Variation in Lexical Stress Productions?

Our final comparison was between L1ENG.ENG, L1ENG.WEL, L1WEL.ENG, and L1WEL.WEL. These tests compared the English and Welsh of bilingual speakers with English or Welsh as their home language to determine whether home language has an effect on their productions.

**FIGURE 6:**
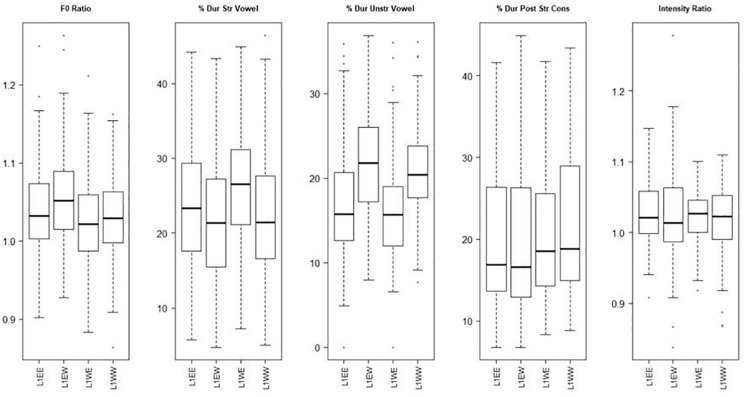
Acoustic correlates of English and Welsh by English-dominant speakers and Welsh-dominant speakers (L1ENG.ENG, L1ENG.WEL, L1WEL.ENG, L1WEL.WEL).

The boxplots (Figure 6) show that the measure that appears to differ most between the two languages is that of the duration of the unstressed vowel, with it taking up a greater percentage of the word in Welsh than in English, but crucially, this appears to be unaffected by home language. In English the stressed vowel takes up a greater percentage, and the unstressed vowel a smaller percentage, than in Welsh. This again shows that in Welsh the unstressed vowel is not shortened. The statistical results in Table 8 support this, with unstressed vowel duration being the only measure with a significant difference emerging (Table 9 shows the means). Table 10 shows the pairwise results for this measure. This shows that all groups differentiate their languages in terms of the duration of unstressed vowels (L1ENG.ENG differs from L1ENG.WEL, and L1WEL.ENG differs from L1WEL.WEL.). The lack of a difference between the groups based on their home language shows that home language has no significant effect on the production of lexical stress.

**TABLE 8:** Results of the multivariate regression analysis comparing L1ENG.ENG, L1ENG.WEL, L1WEL.ENG, and L1WEL.WEL.

Measure		β	*SE*	*t*	*p*
F0	Intercept	1.041	0.005	230.906	<0.001
	L1ENG.WEL	0.0157	0.006	2.527	0.012
	L1WEL.ENG	–0.018	0.007	–2.683	0.008
	L1WEL.WEL	–0.010	0.006	–1.662	0.097
Dur_SV	Intercept	24.389	0.664	36.724	<0.001
	L1ENG.WEL	–2.432	0.910	–2.672	0.008
	L1WEL.ENG	1.498	0.970	1.545	0.123
	L1WEL.WEL	–2.090	0.917	–2.280	0.023
Dur_USV	Intercept	16.814	0.460	36.546	<0.001
	L1ENG.WEL	4.820	0.631	7.644	<0.001
	L1WEL.ENG	–0.715	0.672	–1.064	0.288
	L1WEL.WEL	4.220	0.635	6.646	<0.001
Dur_PSC	Intercept	19.869	0.693	28.666	<0.001
	L1ENG.WEL	–0.182	0.950	–0.191	0.848
	L1WEL.ENG	0.732	1.013	0.722	0.47
	L1WEL.WEL	1.654	0.957	1.729	0.08
Intensity	Intercept	1.027	0.004	276.274	<0.001
	L1ENG.WEL	–0.006	0.005	–1.143	0.253
	L1WEL.ENG	–0.003	0.005	–0.590	0.555
	L1WEL.WEL	–0.008	0.005	–1.527	0.127

**TABLE 9:** Mean values for each measure for Comparison 3 by language group.

Measure	L1.ENG.ENG	L1.ENG.WEL	L1.WEL.ENG	L1.WEL.WEL
F0 Ratio	1.038	1.057	1.020	1.030
Dur_SV	23.656	21.818	25.779	22.324
Dur_USV	17.061	21.793	15.933	21.012
Dur_PSC	19.693	19.901	20.719	21.523
Intensity	1.028	1.022	1.023	1.019

**TABLE 10:** Results of the pairwise *lsmeans* test comparing L1ENG.ENG, L1ENG.WEL, L1WEL.ENG, and L1WEL.WEL.

Measure		β	*SE*	*t*	*p*
Dur_USV	L1ENG.ENG - L1ENG.WEL	–4.820	0.631	–7.644	<0.001
	L1ENG.ENG - L1WEL.ENG	0.715	0.672	1.064	0.712
	L1ENG.ENG – L1WEL.WEL	–4.220	0.635	–6.646	<0.001
	L1ENG.WEL - L1WEL.ENG	5.536	0.653	8.481	<0.001
	L1ENG.WEL - L1WEL.WEL L1	0.600	0.614	0.977	0.762
	WEL.ENG - L1WEL.WEL	–4.936	0.657	–7.512	<0.001

## Discussion

This study investigated the acoustic correlates of lexical stress produced by monolingual and bilingual adolescent males attending the Sixth Form of a bilingual school in Carmarthenshire, West Wales. The study sought to address three main issues. First, we aimed to determine how monolingual speakers of Welsh English realize lexical stress and to what extent their production differs from Welsh on the one hand and SSBE on the other. This would indicate to what extent the contact situation found in this community has affected lexical stress production in Welsh English. Secondly, we aimed to investigate whether monolingual and bilingual speakers from the same community differ in their production of the lexical stress correlates when speaking English. Finally, we aimed to determine the role of home language in the realization of lexical stress in both languages. Below, we discuss the implications of our findings.

### Welsh English in a Contact Situation

To identify the effect of long-term contact on lexical stress production, we systematically compared lexical stress correlates of monolingual speakers of Welsh English to those produced in Welsh by Welsh-English bilinguals from Welsh-speaking homes living in the same community, and to the English realizations produced by monolingual SSBE speakers. As expected from prior studies (Williams, 1983, 1985, 1999), the results revealed that lexical stress is realized differently by speakers of SSBE than by the speakers from the town of Ammanford in Carmarthenshire. SSBE was found to differ from both Welsh and Welsh English on *all* lexical stress measures. The two groups of speakers from Ammanford, however, showed a rather different picture. While there appear to be some differences in acoustic correlates between Welsh and monolingual Welsh English spoken in this area, these are outnumbered by the number of correlates that do not significantly differ between the two languages. In particular, monolingual Welsh English was found to be similar to Welsh on the measures of stressed vowel duration, PSC duration and the f0 difference between stressed and unstressed vowels. This confirms earlier descriptive reports that Welsh English is much like Welsh in that it features a lengthening of the PSC and an unreduced unstressed syllable which may carry a pitch accent and “may be as strong phonetically as the stressed one” (Walters, 2003, p. 220)^4^. Our results only showed differences between monolingual Welsh English and Welsh in the measures of unstressed vowel duration and the intensity difference between stressed and unstressed vowels. While for the former measure monolingual Welsh English clearly aligns with SSBE, for the latter it is intermediary between Welsh and SSBE.

These results suggest that some convergence between Welsh English and Welsh has occurred with regards to lexical stress. While in the absence of any historical data we cannot be entirely sure that this merging is a result of long-term contact, the situation of mass acquisition of L2 English in this area along with many other reported findings of accentual features that are similar in Welsh and Welsh English in Wales (Wells, 1982; Collins and Mees, 1990; Mees and Collins, 1999; Walters, 1999, 2001; Penhallurick, 2004; Mayr et al., 2017) suggests that the observed changes in lexical stress correlates are likely to be contact-induced. This conclusion is reinforced by the fact that prior investigation of other areas of pronunciation produced by the same speakers as in our study (see Mayr et al., 2017) revealed a similar degree of phonetic overlap between Welsh and Welsh English in Carmarthenshire. Specifically, Mayr et al. (2017) found a high degree of phonetic convergence (in their case in the vowel productions) of southern Welsh and Welsh English, along with a few language-specific patterns^5^. In light of this, we suggest that this phonetic overlap is likely to be caused by the continued co-existence of Welsh and Welsh English in the community, resulting in cross-language convergence.

### Monolingualism Versus Bilingualism

Our second research aim was to establish whether monolinguals and bilinguals from the same community differ in their realization of lexical stress correlates when speaking English. That is, does this type of individual linguistic experience play a role in how lexical stress is realized? In order to address this, the realizations of the various acoustic correlates of the bilingual and monolingual speakers were compared. Our study found no differences in any of our acoustic measures between the monolingual and bilingual groups. This is against expectation, as bilingual speech production is generally reported to differ from monolingual speech production in many areas of pronunciation (e.g., Flege and Eefting, 1987; Flege, 1995; Kehoe, 2002, for segments; e.g., Queen, 1996; Elordieta and Calleja, 2005; Mennen et al., 2014, for prosody). In contrast, the present study shows that it does not matter whether a speaker has experience with just one or both languages in the community, the outcome for lexical stress production in English will be the same.

The lack of differences found between the monolingual and bilingual speakers might be partly explained by the cross-language phonetic convergence outlined above. Such convergence was not found across all measures, however, and it therefore still remains to be seen why there is no evidence of cross-language influence in the speech of bilingual speakers on the measurements of unstressed vowel duration and intensity difference between stressed and unstressed vowels. A possible explanation for this finding is that, in this community, these aspects of lexical stress do not function as markers of linguistic experience signaling that the speaker is a speaker of Welsh or not [as is the case for linguistic features in other varieties of Welsh English discussed in Mayr et al. (2019b)]. As extensively discussed in Mayr et al. (2017), the adolescents that participated in this study were part of a close-knit and homogeneous peer group, where the preferred language of interaction was English, and membership of the group was not determined by the ability to speak Welsh. In a similar community in north Wales, Morris (2013) found no evidence of transfer in the production of/r/in bilinguals’ English with all speakers adhering to English norms. The results of the present study therefore provide further evidence that possible transfer effects may be inhibited by other factors such as community or regional norms or peer-group identity. As Mayr et al. (2017, p. 261) conclude, “the effects of linguistic experience can be overridden under certain circumstances, and […] one of these may be a highly homogeneous peer group with shared values and social practices.”

### Home Language

Our final aim was to investigate the influence of home language on the realization of lexical stress correlates. In order to determine this, we compared the production of lexical stress correlates in two groups of bilingual speakers: Welsh-English bilinguals with home language Welsh, and Welsh-English bilinguals with home language English. Both groups of bilinguals were found to differentiate their languages in terms of the duration of unstressed vowels. For intensity, however, the two groups showed merged values for both languages. Moreover, no effect of home language was found for any of the acoustic correlates. These results speak to our third research question, suggesting that home language does not exert an influence on the realization of lexical stress.

To some extent, these results may be explained by the fact that certain features of Welsh and Welsh English have become alike, an argument that was also put forward by Mayr et al. (2017). That is, one would only expect any between-group differences to be realized for features that are distinct in the two languages. This is to some extent also the case in the present study. Where clear distinctions between Welsh and Welsh English are found, as is the case for unstressed vowel duration, we do indeed find that bilingual speakers realize these cross-language differences. However, where the cross-language difference is less clear, as is the case for intensity where Welsh English was found to be intermediate between Welsh and SSBE, the bilingual speakers are found not to differentiate between the languages. It is possible that cross-language differences are only realized when they are relatively substantial, whereas intermediate differences may be ignored.

These results contradict findings in most previous studies, which show an effect of home language on language differentiation of various segmental (Simonet, 2010, 2011b, 2014; Amengual and Chamorro, 2015) and prosodic aspects (Simonet, 2011a; Ordin and Mennen, 2017) of speech production. It also contradicts Morris’s (2013) finding that home language influences the production of/r/in both the Welsh and English of bilinguals in a Welsh-dominant area of North Wales. It is possible that this is due to the relatively homogenous nature of the dataset. Simonet’s (2011a) sample contained both Catalan- and Spanish-dominant bilinguals who differed substantially in their language use across all communicative settings (p. 162). Similarly, Ordin and Mennen (2017) included English-dominant bilinguals in their study who had spoken English at home and also attended English-medium education. In contrast, the main difference in language use among the bilingual speakers in the present study is the use of Welsh in the home, with English tending to be dominant in other communicative contexts outside of the classroom.

## Conclusion

This study investigated cross-language interaction in the realization of lexical stress within a situation of language contact in the town of Ammanford, West Wales. In this particular area, monolingual speakers of Welsh English are in close contact with bilingual speakers of Welsh and Welsh English. By investigating the cross-language differences between Welsh, Welsh English and SSBE, and by examining the influence bilingualism and home language exert on the realization of lexical stress correlates, this study was able to shed new light on the respective influences of long-term language contact and individual linguistic experience. The results show that Welsh and Welsh English have become alike in their realization of most lexical stress correlates, suggesting some degree of convergence. Yet, some cross-language differences were also apparent, albeit just in two of the five acoustic correlates (intensity and unstressed vowel duration). The results further indicate that, by and large, neither bilingualism nor home language exerts a major influence on the realization of lexical stress correlates. While this differs from most previous studies on bilingual speech production, Mayr et al. (2017) also reported null effects of individual linguistic experience in the realization of vowels produced by the same speakers as in our study, and Mayr et al. (2019b) showed that listeners found it difficult to differentiate the Welsh English accents of monolinguals and bilinguals from an adjacent area in South West Wales based on their English accent. This shows that the similarities in the varieties of Welsh and Welsh English spoken in this community extend beyond just a single aspect of pronunciation, and that the situation of long-term contact has led to pervasive phonetic convergence that has affected both segmental and prosodic features. This assertion would therefore suggest that early language experience might not always determine speech patterns, particularly among speakers who are raised in the same community and are members of the same peer group. Having said this, it remains to be seen whether this is reflected in the speech of other Welsh-English bilinguals, notably female speakers in view of the fact that all participants in the current study were male and some previous studies have shown speaker gender effects (e.g., Morris, 2013; Ordin and Mennen, 2017) and, more specifically, the extent to which transfer features are used as a marker of linguistic identity in both the majority and minority language. While the bilingual speakers in our study appear to have converged in their realization of acoustic correlates of lexical stress in English and Welsh in words sharing the penultimate stress pattern, more investigation is needed into how these bilinguals realize other English stress patterns and if the factors of home language and dominance contribute to different realizations, or if local norms continue to permeate Welsh English as a result of contact with Welsh.

## Data Availability Statement

The datasets generated for this study will not be made publicly available. We followed our ethics guidelines which allow only for the named researchers to have access to the actual recordings. We can on request provide Excel files of the generated data, but not the original recordings, as no consent was obtained to use this by other researchers than ourselves.

## Ethics Statement

The studies involving human participants were reviewed and approved by the Cardiff School of Health Sciences Research Ethics Committee, Cardiff Metropolitan University, United Kingdom (ethics reference number: 4654). The patients/participants provided their written informed consent to participate in this study.

## Author Contributions

IM: experimental design, data annotation, data analysis, and writing of manuscript. NK: data extraction, data analysis, and writing of manuscript. RM: experimental design and writing of manuscript. JM: data collection and writing of manuscript.

## Conflict of Interest

The authors declare that the research was conducted in the absence of any commercial or financial relationships that could be construed as a potential conflict of interest.

## Footnotes

1 Schwa, for instance, is not unstressed and reduced in Welsh, and can occur in stressed syllables, while it is not allowed in unstressed final syllables (e.g., Bosch, 1996; Hannahs, 2013).

2 This study was carried out in accordance with the recommendations of the Framework for Ethics Approval of Research Projects, Cardiff School of Health Sciences Research Ethics Committee, Cardiff Metropolitan University, United Kingdom. The protocol was approved by the Cardiff School of Health Sciences Research Ethics Committee, Cardiff Metropolitan University, United Kingdom (ethics reference number: 4654). All subjects gave written informed consent in accordance with the Declaration of Helsinki.

3 The study was confined to males due to ease of availability of participants from male-only peer groups. Moreover, since the data reported here are part of a larger project that also includes acoustic measures of vowel productions (c.f. Mayr et al., 2017), limiting the study to male participants precluded the need to normalize raw Hertz data to account for physiological differences.

4 The lack of reduction of the unstressed ultimate syllable in the speakers in Wales can clearly be seen in Figure 3. It is widely accepted that f0 gradually declines over the course of an utterance, a phenomenon referred to as declination (Cohen and Hart, 1967; Ladd, 1984). As a result, f0 tends to be lower at the end than at the beginning of an utterance. Listeners use this expectation of a gradually declining f0 for normalization purposes, such that when two stressed syllables are perceived as equal in pitch, the second is in fact lower (Pierrehumbert, 1979). When speakers start a new utterance or intonation phrase they tend to ‘reset’ their f0 to a higher level (’t Hart et al., 1990). An f0 reset can also be used to convey semantic information. For instance, speakers use this interruption of an expected global f0 decline to emphasize or stress a word or syllable (e.g., VaissieÌre, 1983; Calhoun, 2010). The f0 reset observed in the speakers in Wales suggests that the unstressed syllable is not reduced and may even be perceived as equal in pitch (given the tendency for listeners to normalize for declination effects in perception) to the stressed syllable. Our findings that there is no difference in average f0 between stressed and unstressed syllables confirms the lack of difference in acoustic salience between stressed and unstressed syllables in Welsh and Welsh English, and confirm earlier claims that f0 does not function as a correlate of lexical stress in Welsh (Williams, 1983).

5 Given that Welsh is a fixed-stress language with no stress-related minimal pairs, it has little need for acoustic salience of the stressed syllable. Hence, there is only a need to signal stress acoustically in English, and this may explain why only language-specific differences were found.
